# *Rickettsia rickettsii* isolation from naturally infected *Amblyomma parvum* ticks by centrifugation in a 24-well culture plate technique

**Published:** 2013-09-20

**Authors:** K. Dzul-Rosado, G. Peniche-Lara, R. Tello-Martín, J. Zavala-Velázquez, R. de Campos Pacheco, M.B. Labruna, E.C. Sánchez, J. Zavala-Castro

**Affiliations:** 1 *Regional Research Center “Dr. Hideyo Noguchi”, Autonomous University of Yucatan, Mexico*; 2 *Faculty of Medicine, Interinstitutional Unit of Clinical and Epidemiological Research. Autonomous University of Yucatan, Mexico*; 3 *Federal University of Mato Grosso, Laboratory of Veterinary Parasitology, Department of Basic Science and animal production, Faculty of Agronomy, Animal Medicine and zootechny, Brazil*; 4 *Parasitic Disease laboratory, Department of Preventive Veterinary Medicine and Animal Health, Faculty of Veterinary Medicine and Zootechny, São Paulo University, São Paulo, SP, Brazil*; 5 *Government Health Services, Hospital Agustín O´Horan SSY, Calle Itzaes s/n. Col Centro, Merida Yucatan, Mexico*

**Keywords:** *Amblyomma parvum*, *Rickettsia rickettsii*, Rocky Mountain spotted fever

## Abstract

Rocky Mountain spotted fever is an acute illness caused by *Rickettsia rickettsii* (*R. rickettsii*) and is transmitted by the bite of ticks of the genera *Dermacentor*, *Amblyomma* and *Rhipicephalus*. The illness results in a high mortality rate and may be easily confused with other febrile syndromes. In Yucatan State, Mexico, childhood cases with a high mortality have been reported. In this work we report the isolation of a Mexican *R. rickettsii* strain from a tick egg mass using an alternative method for Rickettsia isolation with 24-well plates. We also identified a potential vector of *R. rickettsii* in the southeast of Mexico, which is *Amblyomma parvum*.

## Introduction

*Rickettsia rickettsii* (*R. rickettsii*) is the causative agent of Rocky Mountain spotted fever (RMSF), an acute febrile illness transmitted by ticks. This illness has been reported in the US and several other countries in Latin America (Treadwell *et al.*, 2000; Zavala-Castro *et al.*, 2006; Favacho *et al.*, 2011; Argüello *et al.*, 2012). In Mexico, RMSF was reported for the first time in the 1930s mainly in the northern states (Bustamante and Varela 1947). In the Yucatan peninsula RMSF has been recognized in 2005, mainly affecting children and causing a high mortality rate (Zavala-Castro *et al.*, 2008).

The importance of the RMSF in our State is that it could be easily confused with other febrile illnesses, thus delaying its accurate diagnosis and treatment. In our region, several endemic febrile diseases such as leptospirosis and other rickettsioses have masqueraded as dengue hemorrhagic fever, which are considered the principal risk factors for human health (Wilson and Chen, 2002; Zavala-Velazquez *et al.*, 2008). Most human cases of rickettsioses reported in Yucatan State are from small towns where humans and animals involved in the rickettsial natural cycle as amplifying hosts such as dogs, rodents and opossums, live in close proximity. In the north of Mexico, *Rhipicephalus sanguineus* has been reported as the primary vector for *R. rickettsii* (Bustamante and Varela, 1947; Eremeeva *et al.*, 2011). Despite the presence of human cases in the Yucatan peninsula, no vectors of the bacteria have been identified so far.

Traditionally, animals have been used for *R. rickettsii* isolation; however, the worldwide tendency is to use fewer animals for research or diagnosis (Piranda *et al.*, 2011; Soares *et al.*, 2012). Culture in embryonated chicken egg yolk sacs has also been used (Cox, 1938). This procedure requires embryonated eggs of 5 to 8 days of age from flocks fed with an antibiotic-free diet, which are often difficult to obtain rapidly without subscribing to the services of an animal supplier and often requires several blind passages to obtain an isolate. Furthermore, they are easily contaminated.

In order to address the critical gaps in the knowledge about the natural cycle of *R. rickettsii* in our area, we focused our efforts on identifying a potential vector. Our goal was to isolate *Rickettsia* from tick eggs and to develop a method for *Rickettsia* isolation using 24-well plates as an alternative to culturing in embryonated chicken egg yolk sacs.

In this work we report the isolation of Mexican *R. rickettsii* from ticks using a new alternative technique for *Rickettsia* isolation based on the traditional shell vial method (Quesada *et al.*, 2006). We also identify *Amblyomma parvum* (*A. parvum*) as not only a host but also as a potential vector of *R. rickettsii* in the southeast of Mexico.

## Materials and Methods

### Tick samples

62 female ticks were collected from 6 domestic dogs in Dzidzantun (46°11W, 23°38′S), a small municipality in Yucatan State where human cases of RMSF have been previously diagnosed. The identity of the tick species collected were determined using taxonomic keys (Guglielmone and Viñabal, 1994).

The ticks were maintained in our laboratory using glass flasks with a mesh top in an incubator at 27°C and 85% relative humidity until oviposition was observed.

### DNA extraction and rickettsial isolation

DNA extraction from tick egg mass was performed using the DNeasy Blood and Tissue kit (QIAGEN, Valencia, CA) by following the manufacturer’s instructions. Polymerase chain reaction (PCR) was used to evaluate a sample of each tick egg mass for rickettsial DNA. Single-step PCR amplification was performed using genus-specific primers for the citrate synthase gene (*gltA*) (Table 1).

**Table 1:** Primers used for Rickettsia diagnosis and species identification

Gene	Primer	Primer sequence	Product length	Reference
17kDa	17kd1fw	5’-GCTCTTGCAACTTCTATGTT-3’	434 pb	Webb *et al*., 1990
	17kd2rv	5’-CATTGTTCGTCAGGTTGGCG-3’		
*gltA*	Rpcs877fw	5’-GGGGGCCTGCTCACGGCGG-3’	380 pb	Regnery *et al*., 1991
	Rpcs1258rv	5’-ATTGCAAAAAGTACAGTGAACA-3’		
*ompB*	Rp330(2)fw	5’-ATGGCTCAAAAACCAAATTTTC-3’	990 - 999 pb	Peniche-Lara *et al*., 2013
	Rp330(2)rv	5’-AGCTCTACCTGCTCCATTATCT-3’		
*ompA*	Rr190.70fw	5’-ATGGCGAATATTTCTCCAAAA-3’	512 pb	Regnery *et al*., 1991
	Rr190.602rv	5’-AGTGCAGCATTCGCTCCCCCT-3’		

The PCR reaction was performed using 250 ng of DNA in a 50 µl reaction mixture containing 25 pmol of each primer, 200 µM (each) dATP, dCTP, dGTP and dTTP (Invitrogen, Carlsbad, CA, USA), 1 U platinum Taq DNA Polymerase (Invitrogen, Carlsbad, CA, USA); 5 µL of 10X PCR buffer (Invitrogen, Carlsbad, CA, USA); 1.5 µL of 50 mM magnesium chloride. PCR was carried out in a Techne model Genius FGEN02TP thermal cycler (Staffordshire ST15 0SA, UK). The PCR products were resolved by 1% agarose gel electrophoresis followed by staining with ethidium bromide. PCR products were visualized using a UV light source.

### Rickettsia isolation and species identification

Tick egg masses were disinfected using iodine-ethanol mixture (Iodine 0.15 M-ethanol 70%) for 15 minutes and rinsed with sterile water several times to remove the disinfectant.

To avoid contamination and to facilitate the management of the plate, Vero cells were grown in the 8 central wells of a 24-well cell culture plate (Corning Incorporated, Corning NY, USA). 50,000 Vero cells were added to each well with Dulbecco`s Modified Eagle Medium (DMEM) supplemented with 10% fetal bovine serum (FBS) (Biowest, Nuaillé, France) and incubated at 37ºC with 5% of CO_2_ for 48 hrs until 95% confluence was achieved.

Approximately 50 to 60 eggs from each PCR-positive tick egg mass were placed in a 1.5 ml tube, 600 µl of sterile brain heart infusion broth was added and the eggs were crushed using a sterile pestle. The Minimum Essential Medium Eagle (MEM) from the wells was removed, and the wells were refilled with 200 µl each of suspension of the tick egg homogenate. The plate was covered with parafilm and centrifuged at 700 x g for 60 min at 22°C. The supernatant was carefully removed and each well was filled with 1 ml of MEM supplemented with 5% Fetal Calf Serum (FCS) (HyClone Laboratories, Inc., South Logan, Utah, USA), 100 U of penicillin, 100 µg of streptomycin and 250 ng of amphotericin B (Sigma Aldrich, St Louis, MO, USA), and incubated at 33°C in an atmosphere of 5% CO_2_.

The medium was changed every 3 days and replaced with 1 ml of MEM without antibiotics and supplemented with 5% of FCS. A single scrap of the cells from the positive wells was examined for infection on days 9 and 15 by Giménez stain (Giménez, 1964) and by PCR using *gltA* primers.

The species identification of the isolated *Rickettsia* was performed using specific primers for 17 kDa protein; citrate synthase (*gltA*); *ompB*, and *ompA* genes (Table 1). DNA extraction was performed using the DNeasy Blood and Tissue kit (Qiagen, Valencia, CA) by following the manufacturer’s instructions. Single-step PCR amplifications were performed using a Techgene, FTGENE20, Techne thermal cycler (Duxford, UK) (Table 1). The reactions were performed with 100 ng of DNA. Three PCR amplicons of each gene from every positive well were fully sequenced and compared with other sequences in the GenBank.

## Results

Six domestic dogs were included in this study to obtain female tick species. A total of 62 ticks were collected. Five dogs had only *A. cajennense* ticks (12, 5, 9, 9, 15 ticks collected respectively) and only one dog had *A. parvum* ticks (12 ticks collected). No coexistence of the tick species was observed. We successfully obtained 15 and 9 tick egg masses from *A. cajennense* and *A. parvum*, respectively.

Using conventional PCR, we identified the presence of *Rickettsia* *spp*. in 18 of the 24 egg masses analyzed (13 from *A. cajennense* from two different dogs, and 5 from *A. parvum*).

We randomly selected 3 egg masses of *A. cajennense* from each dog, and the 5 positive egg masses of *A. parvum* for *Rickettsia* isolation following the protocol described.

**Fig. 1:**
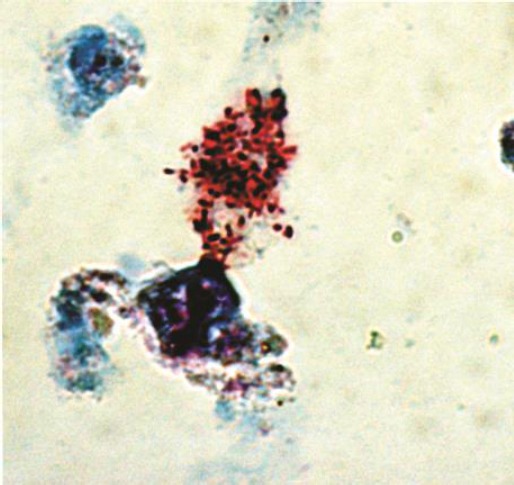
*R. rickettsii* within Vero cells on a cell culture plate after 15 days of incubation, inoculated with a sample from a mass of 50-60 ova of *A. parvum*. (Magnification: 100X).

Numerous red-stained bacteria in the cytoplasm of Vero cells were observed with Giménez staining at day 15 in three of the *A. parvum* egg masses. One scraping of the cells from one positive well was inoculated onto confluent layers of Vero cells in order to establish the isolates in continuous cell culture (Figure 1). No isolation was obtained from any of the *A. cajennense* egg masses used.

After 6 passages, PCR was performed on a scraping from each of the positive wells, and two amplicons of 17 kDa, *gltA*, *ompB* and *ompA* from each well were fully sequenced in the Biomedical Institute of the National Autonomous University of Mexico (IBT-UNAM) using a PRISM Big Dye Terminator Cycle 3.1 Sequencing kit (Applied Biosystems, Foster City, CA) and compared with the previously reported sequences using a Gapped Blast 2.0 (National Center for Biotechnology Information) search of the GenBank database.

The 17 kDa fragment sequence (GenBank accession No. KC464548), citrate synthase (*gltA*) fragment sequence (GenBank accession No. KC469610), *ompB* fragment sequence (GenBank accession No. KC708556) and *ompA* fragment sequence (GenBank accession No. KC763629) showed 100% identity with *R. rickettsii* Sheila Smith strain (GenBank accession No. CP000848.1).

## Discussion

Rocky Mountain spotted fever has been described in almost all countries in Latin America (Labruna *et al.*, 2011). The illness was recognized in Mexico for the first time in the mid-1930s, and recently in more States along the country (Bustamante and Varela, 1947; Martínez-Medina *et al.*, 2007; Eremeeva *et al.*, 2011).

Although its importance for public health has not been determined in Mexico, the variances reported in the morbidity depending on race, ethnicity and differences in the clinical manifestations as reported in Brazil and the United States makes it important to isolate the causative agent in order to make a detailed molecular characterization (Zavala-Castro *et al.*, 2008; Angerami *et al.*, 2009; Dahlgren *et al.*, 2011).

The centrifugation-shell vial technique has been widely employed for the isolation of *Rickettsia* (Quesada *et al.*, 2006). However, in several Latin American countries the acquisition of shell vials is problematic and also expensive. On the other hand, the use of cell culture plates for *Rickettsia* isolation is cheaper and more accessible, and thus allowed us to isolate a *R. rickettsii* strain from infected ticks using the same principle as the shell vial method, but providing a more practical and easy handling technique.

Comparatively, the shell vial technique is beneficial as it allows the isolation quickly than the cell culture technique (5 days vs. 15 days). However, besides the advantage of being more cost effective and easy accessibility of the cell culture plates, our method is highly efficient and is a good alternative for *Rickettsia* isolation and opens up the possibility for other Latin American research groups to obtain the isolates using an inexpensive and efficient technique.

We believe that the use of egg masses is a better strategy to acquire more material for isolation. The experiments to obtain an isolate from *A. cajennense* are currently in progress in our laboratory, as well the molecular characterization of the *R. rickettsii* isolate reported in this study.

It is clear that *A. parvum* resides in domestic and peri-domestic animals in our region. However, its importance in the transmission of the illness to humans is unknown. Interestingly, our present work shows that it is a potential vector for *R. rickettsii* and plays a key role in nature as a host and as a transmitter of *R. rickettsii* to animals, along with other vectors that are responsible for transmitting the disease to humans.

In this work we report a successful isolation of *R. rickettsii* from the egg masses of *A. parvum* by an alternative method using a 24-well plate technique which can be used as an effective substitute for shell vials. In this study, we also demonstrate the potential importance of *A. parvum* as a vector for Rocky Mountain spotted fever in the Yucatan State.
